# Digital Rehabilitation Using Immersive Virtual Reality for Post-COVID Fatigue: A Double-Blind Randomized Controlled Trial

**DOI:** 10.1159/pps/aemag017

**Published:** 2026-09-29

**Authors:** Hannah Lange, Alexander K. Schuster, Stephanie Vock, Wolfgang Eich, Hans-Christoph Friederich, Jonas Tesarz

**Affiliations:** a Department of General Internal Medicine and Psychosomatics, Heidelberg University, Heidelberg, Germany; b Department of Psychosomatic Medicine and Psychotherapy, University Hospital Mainz, Johannes-Gutenberg University Mainz, Mainz, Germany; c Department of Ophthalmology, University Medical Center of the Johannes Gutenberg University Mainz, Mainz, Germany; d Institute for Psychosocial Prevention, Heidelberg University Hospital, Heidelberg, Germany

**Keywords:** Post-COVID fatigue, Virtual reality exercise therapy, Immersive exercise therapy, Neuromuscular rehabilitation, Chronic fatigue syndrome, Randomized controlled trial, Fatigue Severity Scale, Physical activity intervention, Safety and efficacy, Exergaming

## Abstract

**Introduction:**

Post-COVID fatigue is a common and disabling condition with few proven treatments. Immersive virtual reality (VR) exercise therapy may offer a novel rehabilitation approach. We evaluated its efficacy and safety.

**Methods:**

In this single-center, double-blind, parallel-group trial, German adults meeting WHO criteria for post-COVID syndrome, with moderate-to-severe fatigue (Fatigue Severity Scale [FSS] ≥ 36) and no comorbidities better explaining fatigue were randomly assigned (1:1) to a supervised VR exercise therapy or to a supervised conventional exercise therapy. Both interventions comprised 12 sessions of 45 min over 6 weeks. The primary endpoint was change in FSS from baseline to post-intervention, analyzed in the intention-to-treat population (DRKS00032059), and follow-ups took place after 3 and 12 months post-treatment.

**Results:**

Between March 2023 and May 2024, 100 participants were randomized (1:1) to a VR exercise group (VR-EG) or a conventional exercise group (Con-EG). Fatigue severity improved in both groups (VR-EG: –8·29; Con-EG: –5·27), with no significant between-group difference (mean difference: 3.03; 95% CI: –1·02 to 7.06). Only the VR-EG exceeded the established minimal important difference (≥6.3 points). Symptom worsening was less frequent with VR-EG than with Con-EG (9% vs. 23%). Patient Global Impression of Change ratings favored VR-EG (overall improvement: 76.7% vs. 56.5%). Improvements were sustained at 3 and 12 months. Descriptively, women showed greater reductions in fatigue severity than men, especially in the VR-EG. Adverse events were mild and transient and most commonly included headaches, nausea, vertigo, and musculoskeletal pain.

**Conclusion:**

Immersive VR exercise therapy did not significantly outperform an active comparator on the primary endpoint. However, the VR-EG reached the minimal important difference for fatigue at the group level, showed fewer cases of symptom worsening, and reported more favorable global improvement. Considering the limited treatment options available for post-COVID fatigue, these findings indicate a promising clinical signal warranting confirmation in larger, multicenter trials.

## Plain Language Summary

Post-COVID fatigue leaves many people feeling exhausted long after their initial COVID-19 infection. Unfortunately, there are still very few treatments that have been proven to help. In this study, we wanted to find out whether exercise therapy using virtual reality (VR) could be a safe and effective way to reduce fatigue in people with post-COVID. We conducted a study with 100 adults who had post-COVID and experienced moderate or severe fatigue. Half of the participants used a VR exercise program, where they performed movements in a virtual world using a special device (ICAROS). The other half did traditional exercise therapy under supervision. Both groups exercised for 12 sessions over 6 weeks. Both groups felt less fatigued after the treatment. The VR group showed a slightly greater improvement in fatigue compared to the traditional exercise group, but this difference was not large enough to be statistically significant. Only the VR group reached a level of improvement that was large enough to make a meaningful difference in daily life. The improvements lasted for up to 12 months after the treatment. Side effects were mild and temporary, such as dizziness or nausea, which some participants experienced during VR sessions. Our findings suggest that VR exercise therapy could be a promising option for people with post-COVID fatigue. While it did not perform significantly better than traditional exercise in this study, the VR group did show meaningful improvements and fewer side effects. This indicates that VR therapy might be worth exploring further in larger studies.

## Introduction

The COVID-19 pandemic has left a substantial proportion of individuals with persistent symptoms beyond the acute infection, commonly termed long COVID or post-COVID syndrome. Long-COVID generally refers to symptoms that continue or develop beyond 4 weeks after SARS-CoV-2 infection, whereas the World Health Organization (WHO) defines post-COVID syndrome as symptoms that persist for at least 2 months and are still present 3 months after the initial infection without an alternative medical explanation [1]. These sequelae include physical, cognitive, and autonomic impairments. According to the Global Burden of Disease Long COVID Collaborators, 6.2% of those infected with SARS-CoV-2 experienced at least one symptom cluster at 3 months after infection, and 3.2% experienced the symptom cluster “fatigue with bodily pain or mood swings” [2]. Fatigue markedly restricts daily functioning and quality of life and represents a major unmet need in post-COVID care [3–7].

Despite its clinical relevance, mechanisms underlying post-COVID fatigue remain poorly understood. Current treatments are largely symptom-focused and aim to balance physical activity while avoiding overexertion (referred to as post-exertional malaise [PEM]) or treating somatic sequelae of the acute infection [8, 9]. A variety of mechanisms have been proposed to cause post-COVID fatigue such as a dysregulated immune response, persistence of virus (components) or endothelial inflammation and organ damage [10, 11]. Researchers have also put increasing emphasis on neurocognitive and psychosocial aspects of post-COVID fatigue because high psychological burden seems to be a major risk factor for developing post-COVID fatigue [10, 12, 13]. Predictive processing models conceptualize chronic fatigue as a transdiagnostic symptom arising from interactions between sensory input and prior expectations in multiple medical and psychiatric conditions [14]. Even when fatigue is initially triggered by viral or inflammatory processes, these models suggest that maladaptive priors may maintain exhaustion by overriding incongruent sensory input [13]. Thus, fatigue can persist once the acute pathophysiological processes have resolved. Positive sensory prediction errors within virtual reality (VR) – such as reduced effort or enhanced capability – may help recalibrate these expectations.

The ICAROS Health Pro system provides a full-body, VR-based neuromuscular training environment in which users navigate virtual landscapes by shifting their center of gravity. The system consists of a sensor-equipped, body weight-controlled balance platform that supports the user in a prone or semi-prone position, requiring continuous core activation and postural control. Subtle weight shifts and changes in body posture are translated in real time into movement within the virtual environment. By amplifying movement and enabling experiences of ease, speed, or control, immersive VR may recalibrate prior expectations while also enhancing engagement, neuromuscular coordination, and attentional focus [15, 16].

Although VR-based interventions show promise in chronic pain, neurological rehabilitation, and fatigue related to multiple sclerosis or cancer [17–19], evidence in post-COVID fatigue remains scarce. The IFATICO trial was designed as an exploratory, parallel-group pilot study to generate initial evidence on the efficacy and safety of immersive VR exercise therapy in participants with post-COVID fatigue. The primary objective was to assess short-term changes in fatigue severity; secondary objectives examined longer-term outcomes and safety.

## Methods

### Study Design and Setting

This randomized controlled trial was conducted at the Medical Clinic of the University Hospital Heidelberg in Germany, a tertiary care center renowned for its advanced diagnostic and treatment capabilities. Participants attended supervised training sessions at the outpatient clinic of the Department of General Internal Medicine and Psychosomatics. Assessments took place at baseline and directly after the intervention and questionnaires were sent out at baseline, directly after the intervention, and 3 and 12 months after the intervention. To prevent any contamination of intervention masking between the study arms, sessions were delivered in two separate designated clinic areas, one per study arm, minimizing contact between participants in different arms. The full study protocol has been published previously [20], and the prespecified analysis plan is available on the Open Science Framework https://doi.org/10.17605/OSF.IO/23URG. Procedural changes to the study protocol are summarized in online supplementary Table S1 (for all online suppl. material, see https://doi.org/10.1159/pps/aemag017). Patient research partners with post-COVID chronic fatigue contributed to the development of the intervention. This included acceptability testing of the VR procedures and providing feedback that informed refinements to session workflows and participant instructions. The reporting of this study follows the CONSORT guidelines (see online suppl. Table S2).

### Participants

Recruitment took place between March 2023 and April 2024, with follow-up completed in July 2025. Participants were recruited through multiple channels to ensure a broad and representative sample: the outpatient clinic of the Department of General Internal Medicine and Psychosomatics, primary and secondary healthcare networks (including the Long-COVID Network Rhine-Neckar), referrals by attending physicians, and targeted outreach via social media. Informational materials were placed in waiting areas to increase visibility. Recruitment strategies were designed to maximize generalizability. Participants received no formal incentives or financial compensation for completing the study but received therapy free of charge.

Eligibility was evaluated in a standardized, two-step process. First, trained staff (physicians in training using a standardized semi-structured online interview protocol) conducted a telephone screening to verify a medical diagnosis of post-COVID syndrome, the presence of persistent fatigue symptoms, and the participant’s ability to attend study sessions and complete questionnaires and computer-based tasks. Those meeting the initial criteria received a baseline questionnaire assessing fatigue severity using the Fatigue Severity Scale (FSS) [21, 22], gender (asking whether participants identified as male, female, or diverse), comorbidities, and other inclusion-relevant variables like pregnancy or weight over 130 kg (to align with ICAROS safety regulations) and the need for more intensive medical management (e.g., grade 4 on the Post-COVID Functional Scale [PCFS]).

Inclusion criteria required meeting the 2021 WHO clinical case definition of post-COVID-19 condition, including the presence of persistent symptoms attributable to prior SARS-CoV-2 infection [1] that persist for at least 2 months and are still present 3 months after the initial infection without an alternative medical explanation and additionally exhibiting at least moderate fatigue severity (FSS ≥ 36). In line with the WHO definition, laboratory confirmation of the acute infection was not required. No changes to inclusion or exclusion criteria were made after trial initiation. Participants who remained eligible were invited to an in-person assessment at the study site and instructed to avoid strenuous physical activity for 72 h beforehand.

### Interventions

Participants in both groups completed 12 supervised training sessions over 6 weeks (45 min per session). Exercise intensity was monitored using the Borg rating of perceived exertion scale in both groups and adjusted to prevent PEM, following a graded progression within predefined exertion limits. Sessions followed standardized, manualized protocols specific to each group (see online suppl. materials S1, S2) and were supervised by physicians or psychologists in training who received study-specific instruction; adherence was documented in session logs (see online suppl. materials S3, S4). The intervention group received immersive neuromuscular training using the ICAROS Health Pro system, which integrates physical activity with VR. Participants controlled the device and navigated the virtual environment through graded shifts of their center of gravity, with real-time visual feedback delivered via a head-mounted display (see online suppl. Fig. S1). The control group participated in a conventional activation program based on WHO recommendations for post-COVID fatigue, including resistance and low-impact aerobic exercises. Before randomization, both groups received standardized education on neuromuscular control and the relationship between physical activity and fatigue; after randomization, education was tailored to the assigned intervention (see online suppl. materials S5–S7). Both interventions supplemented usual care. Participants were allowed to continue or initiate concomitant treatments, provided these did not violate exclusion criteria, including psychosocial, physiotherapeutic, or occupational therapy. Additional self-management advice followed WHO guidance [8].

### Intervention Integrity and Fidelity

Intervention integrity and fidelity were prospectively monitored using predefined criteria to ensure consistent delivery of core intervention components across study arms. Adherence to session dose, duration, supervision, and manualized content was documented throughout the intervention period. Full fidelity criteria and monitoring procedures are reported in the online supplementary material (see online suppl. material S8).

### Outcomes

The primary outcome measure was the change in fatigue severity from baseline (T0) to post-intervention (T1), as assessed by the FSS [23]. The FSS is a validated tool for evaluating the severity and impact of fatigue, with scores ranging from 9 to 63. This outcome was measured after a standardized 72-h rest period to ensure consistency.

Secondary outcomes included changes in fatigue severity at 3-month (T2) and 12-month (T3) follow-ups and the presence and severity of PEM using the DePaul Symptom Questionnaire (DSQ-PEM) [24]. Physical activity levels were assessed using the Exercise Vital Sign measure [25], while functional outcomes included the six-minute walk test [26] and hand grip strength assessments [27]. Mental capacities were assessed using N-back test [28], Digit Span test [29], and Stroop test [30]. Additional patient-reported outcomes included measures of somatic symptom severity including SSS-8 [31], SSD-12 [32], emotional distress (Hospital Anxiety and Depression Scale) [33], sleep quality using Jenkins Sleep Scale (JSS) [34], and health-related quality of life using the SF12 [35] questionnaire. Global impression of change was evaluated using a patient-reported measure (PGIC) (for detailed description of outcome measures, see online suppl. material S9). An additional secondary endpoint was the fatigue-level responder status at T1. Participants with a reduction in fatigue severity of ≥ 6.3 points on the FSS were classified as responders. This cutoff is consistent with current recommendations for minimal important differences for fatigue patient-reported outcome measures [36–38]. Predefined safety outcomes were the incidence, severity, and relatedness of adverse events during the intervention period, including episodes of PEM. Severity of adverse events was graded by treatment providers as mild (short term, of mild intensity, and not requiring treatment), moderate (medium to long term, of moderate intensity, inhibiting the normal lifestyle; for example, PEM over 14 h), and severe (life-threatening situations, the need for admittance to a hospital, or temporary or permanent disability). These outcomes were assessed through open questions about the days following the last session at the beginning of each following session. This also captured adverse events that did not occur immediately after the last session, which is common for PEM. Specific symptoms characteristic of VR-related discomfort (the “VR triad”: vertigo, motion sickness, fear-related reactions) were proactively monitored and recorded at every session in the intervention arm. All primary and secondary outcomes were prespecified, described, and referenced in the registered study protocol (https://doi.org/10.17605/OSF.IO/23URG).

### Sample Size Calculation

Based on prior data from intervention studies in post-COVID populations [39], a mean difference of 0.9 (SD = 1.5) in FSS scores between groups was anticipated (the study used an item mean instead of an item sum, resulting in an FSS scale of 1–7). A sample size of 45 participants per group provided 80% power to detect this difference with a significance level of 0.05 using a two-sided *t*-test. To account for an estimated 10% dropout rate, the target sample size was set at 100 participants, with 50 in each group. No interim analyses were conducted during the trial.

### Randomization and Masking

Participants were randomly assigned to either the VR exercise group (VR-EG) or the conventional exercise group (Con-EG) in a 1:1 ratio using a secure, web-based randomization system (Sealed Envelope; https://www.sealedenvelope.com/). Block randomization with variable block sizes was employed, stratified by age and baseline fatigue severity. Allocation concealment was maintained throughout the study to minimize selection bias. Randomization was conducted by an independent staff member (S.V.) from the Data Monitoring Board, who was not involved in recruitment or assessments and who had no direct contact with participants. Study personnel involved in enrollment had no access to the allocation sequence at any point.

At enrollment, participants were informed that they would receive one of two innovative neuromuscular training-based intervention programs, without any explicit reference to VR. This information strategy was chosen to minimize expectancy effects and potential disappointment bias associated with allocation to the control condition. Following randomization, only participants assigned to the VR-EG were informed about the VR-based nature of their intervention. To further reduce the risk of unblinding, training sessions for the VR group were conducted in a separate area, and participants were instructed not to discuss their training modality with other participants. Outcome assessors remained blinded by conducting all evaluations in a dedicated assessment room, and participants were repeatedly asked to refrain from sharing details of their training experience. In cases of partial unblinding (*n* = 3), such events were documented (e.g., participants inferring their group allocation), and sensitivity analyses were performed excluding these participants.

### Data Collection and Management

Data were collected at baseline, post-intervention, and at 3-month and 12-month follow-up using validated questionnaires and performance-based assessments. Data handling followed Good Clinical Practice, with secure pseudonymized storage overseen by an independent data manager not involved in trial planning or delivery, with monitoring at predefined recruitment milestones (50%, 75%, and 100%) to ensure data integrity.

### Statistical Analysis

The primary endpoint was analyzed according to the intention-to-treat principle using a linear mixed-effects model (LMM) fitted with restricted maximum likelihood estimation. The model included group, time, their interaction, and the stratification variable age as fixed effects and a random intercept for each participant to account for within-subject correlation. Missing data were not imputed because LMMs can validly handle incomplete repeated measures, and the proportion of missing observations was small (<7%). Mixed-effects model fixed effects were tested using Satterthwaite-approximated degrees of freedom (lmerTest). Confidence intervals for fixed effects were obtained using profile likelihood. We performed standard diagnostic checks for LMMs.

Sensitivity analyses were prespecified to evaluate the robustness of the primary outcome analysis [19]. A per-protocol analysis was conducted including only participants who adhered to all intervention integrity criteria, examining the effects of the intervention under optimal compliance. Additionally, a complete-case analysis was performed, restricting the dataset to participants with no missing values for the primary endpoint to explore potential biases due to missing data. Another sensitivity analysis excluded participants whose group allocation may have been unmasked during the trial, providing insights into the potential impact of unmasking on the observed outcomes.

All secondary outcomes were analyzed exploratively, analogous to the primary outcome using an LMM for continuous variables, and a general mixed-effects model for categorical variables, with no adjustments for multiple testing. Ninety-five percent confidence intervals for model fixed effects were reported to contextualize the clinical relevance of findings [40]. In additional exploratory analyses, gender and fulfillment of PEM diagnostic criteria were added as interaction terms in the mixed-effects model and we conducted subgroup analyses stratified by gender and PEM status. All statistical analyses were performed using R (version 4.4.2; Team, 2024), with a prespecified analysis plan registered prior to data analysis (https://doi.org/10.17605/OSF.IO/23URG). Between-group differences in the proportion of responders, worsening of fatigue, and improvement on the PGIC scale were analyzed post hoc using chi-squared tests.

## Results

### Participant Population

A total of 183 participants were screened for eligibility, resulting in the randomization of 100 participants into two study arms. Specifically, 48 participants were allocated to the VR-EG, while 52 participants were assigned to the Con-EG. In each group, two participants were excluded after randomization: one due to post-randomization identification of an exclusion criterion and one due to scheduling conflicts that precluded participation in the allocated intervention (see Fig. 1). Randomization achieved similar distributions of key variables (e.g., age, gender, and baseline FSS), indicating comparability of the two groups (see Table 1). The overall dropout rate was 15%, with three participants in the VR-EG and 12 participants in the Con-EG discontinuing participation prior to the post-intervention assessment (see Fig. 1; online suppl. Table S3).

**Fig. 1.**
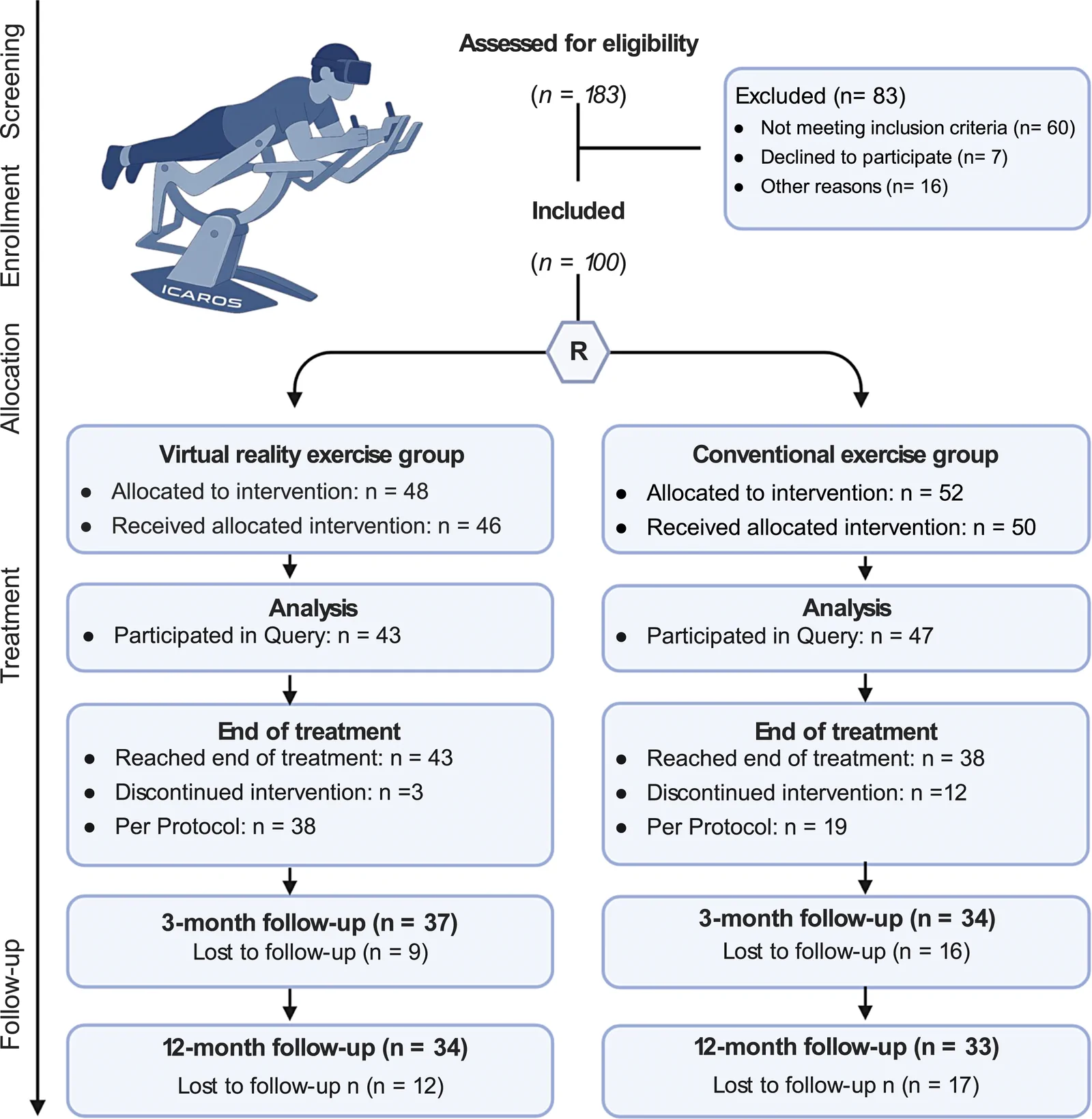
CONSORT flow of participants through the trial. Among the 183 participants assessed for eligibility, 100 met inclusion criteria and were randomized (48 VR-EG, 52 Con-EG). Of these, 46 in the VR-EG and 50 in the Con-EG received the allocated intervention. In the VR-EG, 3 participants discontinued the study and 12 participants in the Con-EG. The primary reasons for dropout included dissatisfaction with intervention (*n* = 1, VR-EG; *n* = 11, Con-EG), personal reasons (*n* = 1 in each group), and no show without given reason (*n* = 1, VR-EG). All dropouts occurred before completion of the T1 assessment.

**Table 1.** Baseline characteristics of the intention-to-treat population

​	VR-EG (*n* = 46)	Con-EG (*n* = 50)
Age	46.5 (14.3)	46.0 (13.6)
Gender: women^a^	38 (83)	35 (70)
In steady relationship	21 (45.7)	30 (60.0)
Actively smoking	1 (2.2)	0 (0)
(Self)employed	44 (95.7)	45 (90.0)
Days of sick leave during the last 3 months^b^	54.6 (42.5)	56.4 (40.2)
BMI	25.3 (5.10)	26.6 (6.10)
Physical performance		
Muscular strength: hand grip ratio	1.14 (0.09)	1.23 (0.51)
Aerobic/functional endurance (6-min walk test)	484 (119)	491 (105)
Cognitive performance		
Working memory capacity (Digit Span test)	14.6 (3.48)	14.8 (3.78)
Working memory processing – false alarms (N-back test)	7.82 (15.9)	5.27 (7.25)
Working memory processing – hits (N-back test)	74.5 (26.6)	74.6 (23.9)
Cognitive interference control (Stroop test [ms])	117 (139)	123 (130)
Somatic symptom burden		
Fatigue (FSS)	56.3 (6.00)	55.8 (6.29)
Presence of PEM (DSQ-PEM)	43 (93.5)	48 (96.0)
Somatic symptom disorder severity (SSD-12)	37.8 (8.44)	38.4 (7.32)
Somatic symptom burden (SSS-8)	15.4 (5.35)	14.8 (4.89)
Weekly physical activity level (EVS)	328 (453)	183 (180)
Sleep problems (JSS)	11.6 (5.30)	12.7 (3.98)
Anxiety symptoms (HADS-A)	6.80 (4.25)	7.54 (3.76)
Depression symptoms (HADS-D)	8.67 (3.45)	8.12 (3.71)
Global emotional distress (HADS total)	15.5 (7.01)	15.7 (6.55)
Physical health (SF12-physical)	29.5 (9.35)	30.8 (7.38)
Mental health (SF12-mental)	42.4 (10.7)	39.6 (10.3)
Duration of post-COVID symptoms > 6 months	38 (82.6)	44 (88.0)

Data are m (SD) or *n* (%) of raw data. Cognitive measures: Digit Span (WAIS-IV), N-back performance (false alarms, hits), and Stroop effect (ms). Six-minute walk test was performed according to a standardized protocol.

BMI, body mass index; PEM, post-exertional malaise; SSD-12, Somatic Symptom Disorder-B Criteria Scale; SSS-8, Somatic Symptom Scale-8; EVS, Exercise Vital Sign; JSS, Jenkins Sleep Scale; HADS, Hospital Anxiety and Depression Scale; FSS, Fatigue Severity Scale; SF12, 12-Item Short Form Health Survey.

^a^ All participants that did not report identifying as a woman, identified as a man, as no participant reported identifying as diverse.

^b^ Days covered by a doctor’s sick note, including weekends and public holidays.

### Participant Population for Sensitivity Analyses

For the complete-case analysis, 43/46 (93.5%) participants in the VR-EG and [47/50 (94.0%)] in the Con-EG had no missing data for the primary endpoint. Outcome assessors were only unblinded in 7/83 (8.4%) of outcome assessments, ensuring the integrity of outcome evaluations. In the standardized post-intervention interview, 2/36 (5.6%) of participants in the VR-EG and 1/40 (2.5%) in the Con-EG correctly identified the content of the other intervention arm (not including missing data).

### Fatigue Severity

Fatigue severity improved descriptively from T0 to T1 in both study groups (see Fig. 2). In the VR-EG, the mean reduction in FSS score (model marginal means) was 8.29 (*p* < 0.0001), whereas the Con-EG showed a mean reduction of 5.27 (*p* = 0.0004). The model effect estimates for the between-group difference in change scores did not reach statistical significance (3.03; 95% CI: –1.02 to –7.06; *p* = 0.15). Only the VR-EG exceeded the predefined clinically relevant reduction of at least 6.3 points. A clinically relevant improvement of ≤ -6.3 points on the FSS was achieved by 17 of 43 participants (39.5%) in the VR-EG and 16 of 47 participants (34%) in the Con-EG (χ^2^ test, *p* = 0.59). All model diagnostics supported the adequacy of the primary analysis (see online suppl. Fig. S2). Sensitivity analyses – including complete-case analysis and exclusion of unmasked participants (VR-EG: *n* = 2; Con-EG: *n* = 1) – were consistent with the primary findings and confirmed the robustness of the results (see online suppl. Table S4). Symptom worsening occurred in 4 of 43 participants (9.3%) in the VR-EG and in 11 of 47 participants (23.4%) in the Con-EG (χ^2^ test, *p* = 0.073), indicating a numerically lower rate of reported deterioration in the VR arm (see online suppl. Tables S5 and S6; online suppl. Fig. S3).

**Fig. 2.**
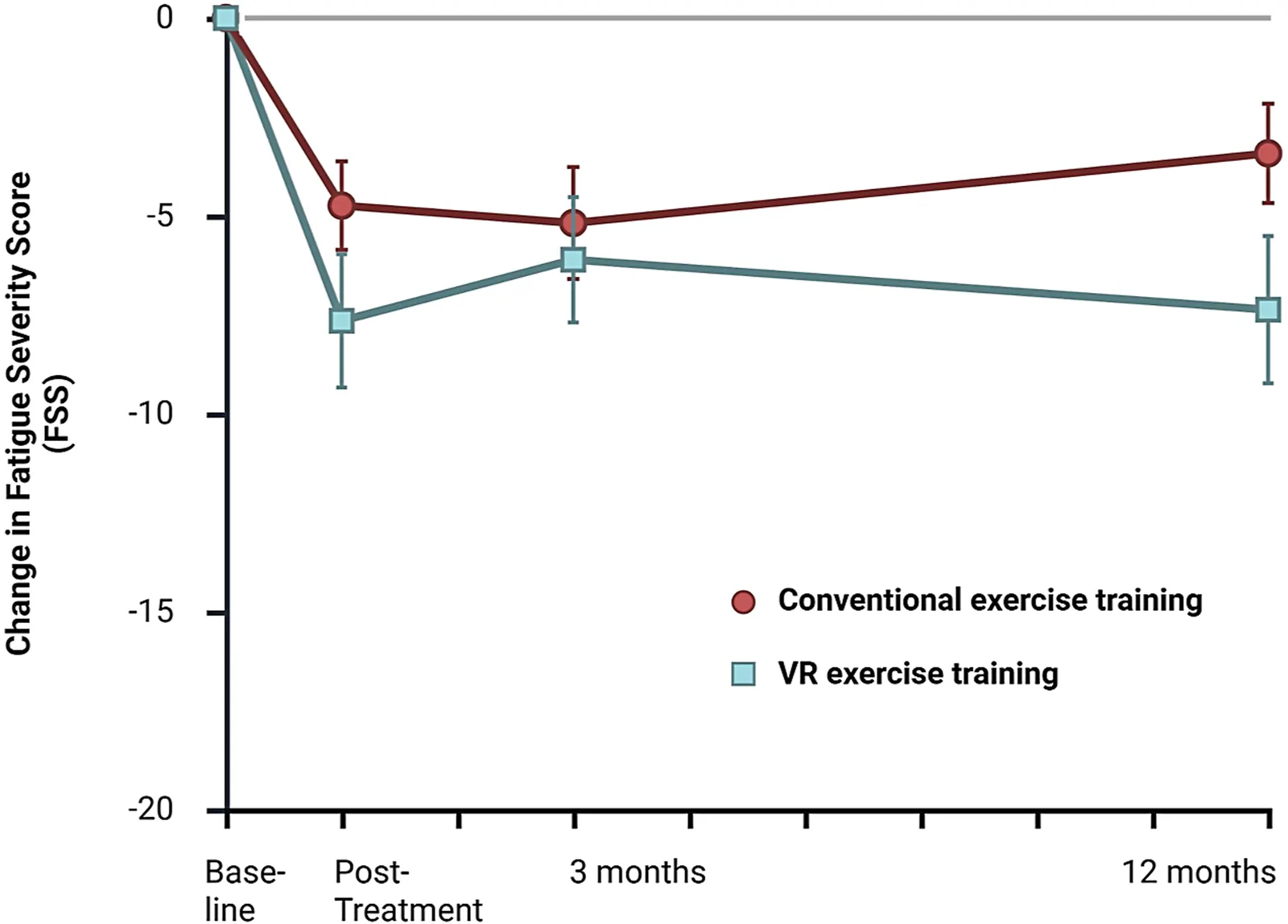
Change in Fatigue Severity Scale scores from baseline to post-intervention and follow-up. Data are mean change in FSS (SEM) of VR-EG (blue) and Con-EG (red) from baseline to post-treatment assessment (6 ± 2 weeks after enrollment), to follow-up 1 (3 months after post-treatment assessment) and to follow-up 2 (12 months after post-treatment assessment). FSS, Fatigue severity score. The horizontal black line indicates no change from baseline (ΔFSS = 0).

### Patients’ Global Impression of Change

Patient-perceived improvement was higher in the VR-EG. In the VR intervention arm, 18 (41.9%) reported strong or very strong improvement and three participants (7.0%) reported feeling slightly or much worse. In the Con-EG, 14 (30.4%) reported strong or very strong improvement and nine participants (19.6%) reported feeling slightly or much worse. Thus, a higher proportion of participants perceived improvement and a lower proportion perceived worsening in the VR-EG than in the Con-EG (see online suppl. Tables S7 and S8).

### Adherence to the Study Protocol

Adherence to the prescribed intervention dose was high in both study groups when excluding dropouts. All participants who completed the study attended at least 10 and no more than 14 of the 12 scheduled sessions. The VR-EG completed a mean of 12.0 (SD: 0.98) sessions, and the Con-EG completed a mean of 11.84 (SD: 0.75) sessions.

Adherence to the prescribed session duration showed notable differences between groups. All participants in the VR-EG exercised for at least 30 and no more than 60 min in ≥80% of sessions, whereas only 21 participants (55.3%) in the Con-EG met this benchmark. Participants who did not meet the duration criterion typically fell below the 30-min threshold in more than 80% of their sessions. Mean session duration was approximately 49 min in the VR-EG and 40 min in the Con-EG. Despite shorter duration, the Con-EG reached a higher mean perceived exertion (RPE: 5.64) than the VR-EG (RPE: 3.61), exceeding the predefined upper limit for exertion (allowed mean RPE: 5.45 vs. observed 5.64).

Fidelity to the manualized intervention content was high. All 43 participants (100%) in the VR-EG and 36 participants (83%) in the Con-EG achieved a mean fidelity score of ≥80% (see online suppl. Table S9).

### Adverse Events

Adverse events were infrequent and generally mild across both study arms. In the VR-EG, 28 participants (60.9%) experienced at least one adverse event during the study, corresponding to 66 events across all VR sessions (12.6%). The most common events were symptoms from the VR triad – motion sickness (19 participants, 41.3%), vertigo (40 participants, 87%), and mild fear-related responses (16 participants, 34.8%) – which were actively and systematically queried at each session. These symptoms were transient and resolved within the session. In the Con-EG, adverse events were rare, with only two participants (4%) reporting any event across the entire intervention period. No severe adverse events occurred in either group. Vertigo, nausea, and headaches lasting beyond the end of the treatment session were the most frequently reported mild or moderate adverse events. Episodes of worsening PEM were reported three times in the VR-EG and once in the Con-EG. No participant discontinued the study due to adverse events in either arm (see Table 2, for descriptive overview of adverse events, see online suppl. Fig. S4).

**Table 2.** Adverse events in the VR-EG and the Con-EG

Patient reported	VR-EG		Con-EG	
	Sessions (*n* = 525), *n* (%)	Participants (*n* = 46), *n* (%)	Sessions (*n* = 479), *n* (%)	Participants (*n* = 50), *n* (%)
Types				
Related to Intervention	26 (4.9)	22 (47.8)	0 (0)	0 (0)
Possibly related to intervention	40 (7.6)	18 (39.1)	2 (0.4)	2 (4)
Unrelated to intervention	1 (0.2)	1 (2.2)	0 (0)	0 (0)
Total	66 (12.6)	28 (60.9)	2 (0.4)	2 (4)
Severity				
Mild	54 (10.2)	26 (56.5)	0 (0)	0 (0)
Moderate	13 (2.5)	9 (19.6)	2 (0.4)	2 (4)
Severe	0 (0)	0 (0)	0 (0)	0 (0)
Total	66 (12.6)	28 (60.9)	2 (0.4)	2 (4)

Assessed by trainer	Events, *n* (%)	Participants, *n* (%)	Severity, m (SD)
VR triad			
Motion sickness	72 (13.7)	19 (41.3)	3.3 (1.82)
Vertigo	217 (41.3)	40 (87)	3.51 (1.94)
Fear	35 (6.7)	16 (34.8)	3.46 (1.95)

Adverse events are reported as total events (percentage of all supervised sessions) and number of participants affected (percentage of all participants). Events were classified as related, possibly related, or unrelated to the intervention and graded for severity (mild, moderate, severe). VR-specific symptoms (the “VR triad”: motion sickness, vertigo, fear-related reactions) were proactively monitored and recorded by therapists at every VR session; severity ratings reflect a 0–10 numerical rating scale. No severe adverse events occurred in either study arm. During one training session and for several participants, we documented multiple adverse events. Therfore, the total number of training sessions/ participants with adverse events does not correspond to the sum of the sub-categories.

### Fatigue Severity at Follow-Ups

At T2, both groups showed sustained improvement relative to baseline, with a mean reduction in FSS model marginal means of 7.3 in the VR-EG (*p* = 0.0002) and 6.0 in the Con-EG (*p* = 0.0039), while the between-group difference was not statistically significant (−2.07; 95% CI: –7.63 to 3.49; *p* = 0.46). At T3, the VR-EG maintained a mean reduction in model marginal means of 8.04 (*p* < 0.0001), whereas the Con-EG showed a smaller, nonsignificant reduction of 4.04 (*p* = 0.10). The model effect estimates for between-group difference at T3 did not reach statistical significance (−2.08; 95% CI: –7.9 to 3.74; *p* = 0.48).

### Additional Secondary Outcomes

Across additional secondary outcomes, including somatic symptom severity, depression, anxiety, sleep, and cognitive and physical performance, both groups showed small improvements with no significant between-group differences (Table 3). In exploratory moderator analyses, gender was not significantly associated with changes in fatigue severity over time in the overall sample (*p* = 0.09), and this association did not differ between treatment groups (*p* = 0.24). Descriptively, women showed greater reductions in fatigue severity than men. Among women, fatigue severity improved by 9.36 points in the VR-EG and by 5.53 points in the control exercise group (mean difference: 3.83; 95% CI: –1.24 to 8.88). Among men, the corresponding reductions were 2.57 and 4.67 points, respectively (mean difference: −2.09; 95% CI: –7.0 to 2.81), indicating numerically greater improvements in the control exercise group.

**Table 3.** Secondary outcomes at baseline and post-treatment

​	Group (*n*)	T0 – baseline: m (SE)	T1 – post-treatment: m (SE)	Within group: *p* value	Between group: *p* value	Difference in change (CI)/odds ratio
Physical performance						
Muscular strength (hand grip ratio)	VR-EG (46)	1.14 (0.11)	1.35 (0.11)	0.17	​	​
​	Con-EG (50)	1.23 (0.1)	1.15 (0.11)	0.57	​	​
​	​	​	​	​	0.17	−0.3 CI (−0.72 to 0.12)
Aerobic/functional endurance (6-min walk test)	VR-EG (46)	484 (16.7)	524 (17.3)	0.011*	​	​
​	Con-EG (48)	491 (16.3)	517 (17.1)	0.096	​	​
​	​	​	​	​	0.52	−13.94 CI (−56.12 to 28.56)
Cognitive performance						
Working memory capacity (Digit Span test)	VR-EG (45)	14.6 (0.52)	14.8 (0.54)	0.56	​	​
​	Con-EG (50)	14.8 (0.5)	15.8 (0.52)	0.012*	​	​
​	​	​	​	​	0.18	0.71 CI (−0.31 to 1.72)
Working memory processing – false alarms (N-back test)	VR-EG (45)	7.82 (1.5)	5.5 (1.6)	0.22	​	​
​	Con-EG (49)	5.26 (1.43)	5.58 (1.56)	0.86	​	​
​	​	​	​	​	0.32	2.64 CI (−2.48 to 7.77)
Working memory processing – hits (N-back test)	VR-EG (45)	74.5 (3.87)	77.9 (4.1)	0.45	​	​
​	Con-EG (49)	74.6 (3.71)	77.9 (4.01)	0.45	​	​
​	​	​	​	​	0.99	−0.085 CI (−12.37 to 12.12)
Cognitive interference control (Stroop test)	VR-EG (45)	117 (18.1)	109 (19.5)	0.74	​	​
​	Con-EG (47)	124 (17.7)	128 (19.2)	0.85	​	​
​	​	​	​	​	0.71	12.73 CI (−54.62 to 79.82)
Somatic symptom burden						
Presence of PEM (DSQ-PEM)	VR-EG (46)	93.5% (3.6%)	79.1% (6.2%)	0.059	​	​
​	Con-EG (50)	96.0% (2.8%)	89.4% (4.5%)	0.23	​	​
​	​	​	​	​	0.8	Odds ratio: 0.75
Somatic symptom disorder severity (SSD-12)	VR-EG (46)	37.8 (1.31)	36.0 (1.33)	0.094	​	​
​	Con-EG (50)	38.4 (1.25)	36.3 (1.28)	0.034*	​	​
​	​	​	​	​	0.78	−0.4 CI (−3.17 to 2.39)
Somatic symptom burden (SSS-8)	VR-EG (46)	15.4 (0.81)	13.9 (0.82)	0.047*	​	​
​	Con-EG (50)	14.8 (0.77)	14.4 (0.79)	0.6	​	​
​	​	​	​	​	0.28	1.13 CI (−0.92 to 3.18)
Weekly physical activity level (EVS)	VR-EG (46)	324 (54.7)	361 (56.2)	0.54	​	​
​	Con-EG (50)	182 (52.4)	225 (54.3)	0.46	​	​
​	​	​	​	​	0.94	6.01 CI (−155.61 to 167.51)
Sleep problems (JSS)	VR-EG (46)	11.52 (0.7)	9.56 (0.71)	0.0007***	​	​
​	Con-EG (50)	12.69 (0.67)	10.58 (0.69)	0.0002***	​	​
​	​	​	​	​	0.85	−0.15 CI (−1.68 to 1.39)
Anxiety symptoms (HADS-A)	VR-EG (46)	6.79 (0.59)	6.57 (0.6)	0.64	​	​
​	Con-EG (50)	7.54 (0.57)	6.45 (0.58)	0.017*	​	​
​	​	​	​	​	0.18	−0.87 CI (−2.13 to 0.39)
Depressive symptoms (HADS-D)	VR-EG (46)	8.63 (0.58)	7.71 (0.59)	0.044*	​	​
​	Con-EG (50)	8.12 (0.57)	7.26 (0.58)	0.055	​	​
​	​	​	​	​	0.91	0.074 CI (−1.15 to 1.3)
Global emotional distress (HADS total)	VR-EG (46)	15.4 (1.06)	14.3 (1.08)	0.17	​	​
​	Con-EG (50)	15.6 (1.01)	13.7 (1.04)	0.018*	​	​
​	​	​	​	​	0.49	−0.8 CI (−3.05 to 1.46)
Physical health (SF12-physical)	VR-EG (46)	29.5 (1.26)	32.0 (1.28)	0.009**	​	​
​	Con-EG (50)	30.8 (1.2)	30.9 (1.23)	0.9	​	​
​	​	​	​	​	0.07	−2.45 CI (−5.07 to 0.18)
Mental health (SF12-mental)	VR-EG (46)	42.5 (1.6)	45.0 (1.64)	0.09	​	​
​	Con-EG (50)	39.6 (1.54)	43.0 (1.58)	0.019*	​	​
​	​	​	​	​	0.68	0.85 CI (−3.14 to 4.82)

Values for within-group difference are model marginal means (SE). Between-group differences reflect the change from baseline to T1 and are presented as model effect estimates with 95% confidence intervals for continuous outcomes or model odds ratios for binary outcomes. Cognitive outcomes include Digit Span (WAIS-IV), N-back performance (false alarms, hits), and Stroop effect (ms). Six-minute walk test was conducted according to a standardized protocol. *p* values represent within-group changes from T0 to T1 or between-group differences in change from T0 to T1 as indicated.

VR-EG, experimental group (VR exercise group); Con-EG, conventional exercise group (conventional exercise therapy); PEM, post-exertional malaise; SSD-12, Somatic Symptom Disorder-B Criteria Scale; SSS-8, Somatic Symptom Scale-8; EVS, Exercise Vital Sign; JSS, Jenkins Sleep Scale; HADS, Hospital Anxiety and Depression Scale; SF12, 12-Item Short Form Health Survey. *Significant *p*-values, **Moderately significant *p*-values, ***Highly significant *p*-values.

Fulfillment of PEM diagnostic criteria was significantly associated with changes in fatigue severity over time in the overall sample (*p* = 0.003), with participants not fulfilling PEM criteria showing greater improvement. However, this association did not differ between treatment groups (*p* = 0.24). Among participants fulfilling PEM criteria, fatigue severity improved by 4.10 points in the VR-EG and by 3.95 points in the control exercise group (mean difference: 0.15; 95% CI: −2.63 to 2.97). Estimates for participants not fulfilling PEM criteria could not be reliably obtained because the corresponding subgroup was too small and the model did not converge.

## Discussion

This randomized controlled trial evaluated the efficacy and safety of supervised immersive VR exercise therapy compared with supervised conventional exercise therapy in participants with post-COVID fatigue. Both interventions were associated with reductions in fatigue severity. Improvement in the VR-EG exceeded the minimally clinically important difference, but no statistically significant between-group difference was observed on the primary endpoint. VR exercise therapy was feasible in this population and was generally well tolerated, with no serious adverse events or sustained worsening of PEM.

All sensitivity analyses supported the adequacy of results, including the per-protocol analysis correcting for the much higher dropout rate in the comparison group. Secondary analysis on somatic symptom severity, depression, anxiety, sleep and cognitive and physical performance did not show significant differences between groups.

Several exploratory outcomes showed a consistent pattern favoring VR, including lower perceived exertion, higher rates of clinically meaningful individual improvement, more favorable global impressions of change, fewer reports of fatigue worsening, and more consistent regulation of exertion. This may reflect enhanced acceptability and safety of VR exercise interventions. Improvements were largely sustained at 3- and 12-month follow-up, although between-group differences remained statistically nonsignificant, emphasizing cautious interpretation regarding durability.

Effect sizes were modest in both groups, which is unsurprising given the substantial baseline fatigue severity, high symptom chronicity, and the limited treatment exposure of approximately 12 supervised sessions totaling less than 10 h. Symptom chronicity was slightly lower in the VR-EG (82.6% vs. 88% reported having had symptoms for more than 6 months), so chronicity might be a negative predictor for treatment effects, which could point toward a potential benefit of earlier therapeutic intervention. However, given the relatively small difference between groups and the exploratory nature of these observations, this finding should be interpreted cautiously.

In previous literature, evidence-based treatment options for post-COVID fatigue remain limited. Recently, a meta-analysis specifically evaluated pulmonary rehabilitation and supported its potential to improve fatigue in post-COVID syndrome [41]. A systematic review evaluating interventions for long COVID including post-COVID fatigue concluded that cognitive behavioral therapy and combined physical and mental rehabilitation probably improve post-COVID fatigue, whereas evidence for other interventions remains insufficient [42]. The study also found that approximately half of the included studies were at high risk of bias, while one in four trials raised concerns regarding study integrity [42]. In this study, the use of an active comparator, structured supervision, and efforts to maintain masking strengthen internal validity and reduce inflation of treatment effects. In this rigorous setting, the absence of a statistically significant between-group difference highlights the need for adequately powered, multicenter trials to clarify the magnitude and durability of any incremental benefit of immersive VR. Previous VR applications have been associated with reductions in chronic fatigue in patients with multiple sclerosis and cancer survivors [18, 19]. A few studies investigated VR-based exercise interventions for post-COVID fatigue but reported only small or inconsistent effects and were limited by small sample size [43–45]. These studies differed substantially with respect to immersion, exercise modality, treatment dose, and therapeutic rationale, limiting direct comparison with the present findings.

Both interventions were generally well tolerated, with high adherence and minimal adverse events. VR participants most frequently reported transient vertigo or motion sickness, which resolved shortly after sessions and required no medical intervention. No serious adverse events occurred in either group, and no participant discontinued the study because of adverse events. Fatigue worsening at the end of the trial was reported less frequently in the VR-EG than in the Con-EG, although these differences should be interpreted cautiously. They may reflect expectancy effects, disappointment related to prior treatment experiences, or symptom fluctuations independent of the interventions. There was a high degree of symptom chronicity in both groups and a high prevalence of PEM at baseline, but exacerbations of PEM were rare in both study arms, and the proportion of participants meeting diagnostic criteria for PEM decreased over time. Given ongoing concerns regarding physical activation in patients with severe post-COVID fatigue, these findings provide reassurance regarding the safety of both immersive VR and conventional supervised activation programs. This pattern is consistent with recent systematic reviews reporting no evidence of consistent harm from exercise-based interventions in post-COVID populations [46, 47] and one recent scoping review that found VR exercise in post-COVID to be safe, enjoyable, and motivating [48]. Differences in adverse event reporting between groups should also be interpreted in light of more proactive monitoring of VR-specific symptoms in the intervention arm.

The selective improvement in fatigue severity, with limited changes across secondary outcomes, may be consistent with theoretical models linking fatigue to alterations in interoceptive prediction and effort perception. Predictive coding frameworks propose that perceptions of bodily state arise from the integration of sensory input with prior expectations, and that prediction errors update these expectations [14, 49]. In post-COVID fatigue, biased predictions related to exertional threat and limited physical capability may override diffuse sensory feedback [13]. Immersive VR provides a structured multisensory context in which such expectations might be challenged through experiences of reduced effort, enhanced capability, attentional shifts away from fatigue-related sensations, or altered bodily self-perception and embodiment-related experiences. However, these mechanisms were not directly assessed in this trial and treatment effects probably rely on the interplay of a variety of different neurocognitive mechanisms [50]. Restorative environmental exposure, attentional restoration, stress reduction, affective modulation, immersive embodiment-related effects, out-of-body-like experiences, and motivational engagement might also be relevant VR mechanisms [51]. Recent studies investigating VR exercise in post-COVID fatigue suggested potential benefits related to immersive relaxation, distraction, or increased engagement. VR has been widely adopted in rehabilitation, primarily to simulate task-specific activities, provide graded exposure to challenging situations, or increase motivation and engagement through gamification [52, 53]. In contrast, the present intervention combined immersive VR with continuous whole-body control on an unstable platform. Navigation within the virtual environment depended entirely on shifts in body position, requiring continuous postural control, balance, trunk stabilization, and integration of visual, vestibular, proprioceptive, and motor input. These features distinguish the present intervention from previous VR approaches in post-COVID rehabilitation and may engage mechanisms beyond distraction or motivational effects alone. One recent study used non-immersive VR during treadmill exercise [44], whereas another implemented only a single exercise session [45] in a relaxing VR environment. In an interesting approach, one study tried to alter post-COVID patients’ perceived effort and fatigue by giving false respiratory biofeedback in VR (during a bicycle ride, consistent with predictive coding approaches, although some participants reported the experience as disturbing) [43]. The heterogeneity of these interventions with respect to immersion, exercise modality, treatment dose, and therapeutic rationale limits direct comparison between studies. Future trials should incorporate additional control conditions (e.g., passive VR without postural engagement, VR environments without naturalistic content, or matched non-VR natural imagery) to untangle different mechanistic approaches.

This study has several limitations. First, the sample size was modest, and the trial was not powered to detect small or moderate between-group differences, increasing the risk of type II error. Second, although an active comparator and double-blind procedures were used to minimize expectancy effects, complete functional masking is challenging in trials involving immersive VR. Masking was largely successful, with only a small number of participants correctly inferring the alternative intervention arm, but residual unblinding cannot be fully excluded. Third, treatment exposure was brief. While this reflects a realistic early-phase intervention, it may have attenuated effects on secondary, functional, or cognitive outcomes that typically require longer rehabilitation periods. Fourth, safety and tolerability outcomes relied partly on participant self-report and interactions with study personnel, which may be influenced by reporting biases in either direction.

## Conclusion

In summary, this exploratory randomized controlled trial shows that immersive VR exercise therapy is feasible, safe, and acceptable for patients with post-COVID fatigue. Both interventions were associated with reductions in fatigue severity, and the consistent pattern of exploratory outcomes – including higher rates of clinically meaningful improvement, more favorable global impressions of change, fewer reports of symptom worsening, and more consistent exertional regulation in the VR group – suggests a potential clinical signal favoring immersive VR. This signal may be most relevant for patients who struggle with pacing or exertional regulation in conventional rehabilitation settings. Given the limited treatment options available for post-COVID fatigue, these findings support the rationale for larger, multicenter trials with longer follow-up and integrated mechanistic measures to determine the magnitude, durability, and clinical role of VR-based exercise within multimodal rehabilitation pathways for post-COVID fatigue and related fatigue conditions. In addition, future trials should systematically investigate potential dose-response relationships by comparing different training intensities, numbers of treatment sessions, and intervention durations. Such studies may help clarify whether treatment effects are partially dose-dependent and whether findings can be generalized to less supervised and more heterogeneous clinical settings.

## Statement of Ethics

This study was approved by the Medical Faculty of the University of Heidelberg Ethics Committee (S-017/2023) and conducted in accordance with the Declaration of Helsinki. All participants provided written informed consent. The trial was preregistered in the German Clinical Trials Register (DRKS00032059) on June 16, 2023.

## Conflict of Interest Statement

ICAROS GmbH provided the ICAROS device and software free of charge. At an early stage of the project, an advisory meeting with ICAROS GmbH was held to discuss technical aspects relevant to the development of the Virtual Reality intervention (e.g., session frequency and duration). ICAROS GmbH had no further involvement and no role in the study design, outcome selection, data collection, data analysis, data interpretation, decision to publish, or writing of the report. We have no further competing interests.

## Funding Sources

The study was funded by Heidelberg University Hospital. ICAROS GmbH provided the ICAROS device.

## Author Contributions

All authors listed on the manuscript have contributed sufficiently to the project and manuscript, meet the four ICMJE criteria for authorship, and accept accountability for all aspects of the work. J.T. supervised the project and J.T. and H.L. developed the methodology and drafted the manuscript. H.L. collected the data. H.L. and A.K.S. analyzed the data. S.V. was responsible for data monitoring. H.L., A.K.S., S.V., W.E., H.-C.F., and J.T. were involved in interpretation of data and revised the manuscript critically for important intellectual content.

## Data Availability

Individual participant data cannot be shared due to institutional and regulatory restrictions. Aggregated data are available from the corresponding author upon reasonable request, and the statistical code was uploaded to GitHub (https://github.com/hannahsophielange/IFATICO).
